# Immunity against the Obligate Intracellular Bacterial Pathogen Rickettsia australis Requires a Functional Complement System

**DOI:** 10.1128/IAI.00139-18

**Published:** 2018-05-22

**Authors:** Sean P. Riley, Abigail I. Fish, Fabio Del Piero, Juan J. Martinez

**Affiliations:** a Vector-borne Disease Laboratories, Department of Pathobiological Sciences, Louisiana State University School of Veterinary Medicine, Baton Rouge, Louisiana, USA; b Louisiana State University School of Veterinary Medicine, Baton Rouge, Louisiana, USA; Yale University School of Medicine

**Keywords:** Rickettsia, complement receptors, complement resistance, immune evasion, intracellular bacteria, intravascular infections, serum resistance

## Abstract

The complement system has a well-defined role in deterring blood-borne infections. However, complement is not entirely efficacious, as several bacterial pathogens, including some obligate intracellular pathogens, have evolved mechanisms for resistance. It is presumed that obligate intracellular bacteria evade complement attack by residing within a host cell; however, recent studies have challenged this presumption. Here, we demonstrate that the complement system is activated during infection with the obligate intracellular bacterium Rickettsia australis and that genetic ablation of complement increases susceptibility to infection. Interaction of Rickettsia australis with serum-borne complement leads to activation of the complement cascade, producing three effector mechanisms that could negatively influence R. australis. The C9-dependent membrane attack complex can lead to deposition of a bacteriolytic membrane pore on the bacteria, but this system does not contribute to control of rickettsial infection. Similarly, complement receptor (CR1/2)-dependent opsonophagocytosis may lead to engulfment and killing of the bacteria, but this system is also dispensable for immunity. Nevertheless, intact complement is essential for naturally acquired and antibody-mediated immunity to Rickettsia infection. Comparison of infection in mice lacking the central complement protein C3 with infection in their wild-type counterparts demonstrated decreases in gamma interferon (IFN-γ) production, IgG secretion, and spleen hyperplasia in animals lacking complement. The correlation between loss of secondary immune functions and loss of complement indicates that the proinflammatory signaling components of the complement system, and not membrane attack complex or opsonophagocytosis, contribute to the immune response to this pathogen.

## INTRODUCTION

Bacteria in the genus Rickettsia are the etiologic agents of many different diseases of humans and mammals, including typhus and spotted fevers. Infection with various rickettsial pathogens can generate different clinical signs and outcomes, but these infections share similar characteristics, including obligate intracellular parasitism, transmission via arthropods, and endothelial tropism (reviewed in reference 1). While Rickettsia is localized primarily within the infected host cytoplasm, the bacteria likely encounter the extracellular milieu of the bloodstream during introduction into a mammalian host and intravascular dissemination. It is presumably during these transitional stages or during introduction into a potential mammalian host that pathogenic Rickettsia species encounter the host complement system.

The mammalian complement system is a key serum-borne immune mechanism, which consists of a collection of soluble proteins that circulate as inactive precursors. These proteins are activated by proteolysis, conformational changes, or macromolecular assembly through recognition of molecular markers on the surface of foreign cells (2). The various complement activation pathways converge at the formation of active protease complexes called C3 convertases (3). C3 convertases amplify the complement cascade by catalyzing deposition of the C3b opsonin, production of inflammatory anaphylatoxin C3a, and formation of a secondary C5 convertase. The various downstream effects of the complement cascade are all dependent on an intact C3 protein; therefore, genetic ablation of the gene encoding C3 eliminates essentially all complement-dependent effector activities (4).

C3 convertase formation is followed by a proteolytic and amplifying cascade that ultimately results in two antimicrobial phenotypes, deposition of a porelike membrane attack complex (MAC) and opsonization of the recognized particle. In addition to bacterial lysis and opsonization, complement activation leads to production of the proinflammatory anaphylatoxins. Anaphylatoxins stimulate innate immunity by inducting oxidative burst in macrophages, eosinophils, and neutrophils (5–7) and modulate the adaptive immune response to infection (8). Anaphylatoxin signaling has been linked to B-cell activation (9), Th1 polarization (10), and peripheral T-cell survival (11). In this regard, the complement system lies at the intersection of innate and adaptive immunity. Finally, the complement system directly contributes to the adaptive immune response. Following deposition of IgM or several IgG molecules, the classical complement pathway is quickly and robustly activated. Antibody-mediated deposition of the C1qrs complex recruits a stable C3 convertase to quickly and strongly amplify the complement cascade, resulting in a dramatic increase in effectiveness of antibody recognition of an antigen (reviewed in reference 12).

Recent investigations into the interaction between pathogenic Rickettsia species and the host complement system have partially illuminated this ongoing host-pathogen interplay. Analysis of serum from human patients presenting with Mediterranean spotted fever demonstrated a statistically significant increase in the complement activation marker C3a (13). This finding suggests that the complement system is activated *in vivo* during a Rickettsia infection and that complement may play a role in the immune response to this class of pathogens. Conversely, analysis of the interaction between Rickettsia conorii and *in vitro* serum identified a series of molecular interactions that ultimately prevent complement-mediated killing of the bacteria (14–16). These apparently conflicting findings suggest that complement activation in a naive mammal is unlikely to contribute to protective immunity against rickettsial infection.

Here, we have employed a murine model of disseminated Rickettsia australis infection of the C57BL/6J mouse to determine whether individual complement mechanisms play a role in protection from rickettsial infection. *In vivo* analysis of the Rickettsia-complement interaction employs a murine model of disseminated Rickettsia australis infection of the C57BL/6J mouse. R. australis is the only Rickettsia species demonstrated to infect mice with the C57BL/6J background. This infection model induces vascular pathology that is similar to that of other spotted fever group (SFG) Rickettsia infections and in other mammals (17). By employing this specific infection model, we gain access to the powerful genetic tools available in the C57BL/6J mouse to query the roles of complement during Rickettsia infection. Taken together, analysis of R. australis infection and the subsequent immune response of C57BL/6J mice will clarify the host immune response to Rickettsia infection, and achieved results will likely be applicable to other animals and Rickettsia species.

## RESULTS

### The complement system is activated by exposure to R. australis but does not lyse the bacteria.

**FIG 1:**
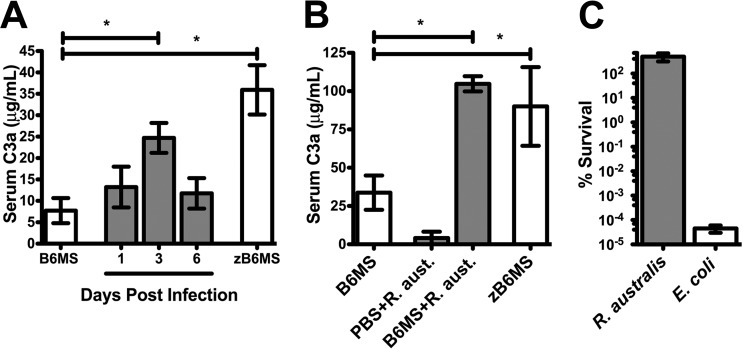
The murine complement system is activated by R. australis exposure but does not affect survival of the bacteria. (A) Concentration of the activated complement protein C3a in normal mouse serum (B6MS), after treatment with the complement activator zymosan (zB6MS), or at specific intervals of a sublethal infection with R. australis (gray). (B) C3a production in *ex vivo* C57BL/6J mouse serum (B6MS), after incubation of purified R. australis with PBS or B6MS, or after zymosan treatment (zB6MS). (C) Survival percentage of R. australis and E. coli DH5α cells after challenge with B6MS. *, *P* < 0.05.

A recent report established that human patients presenting with R. conorii infection have elevated activated serum complement levels (13). To examine if this phenotype occurs in experimental models of infection, we intravenously infected susceptible C57BL/6J mice with a sublethal dose of R. australis (17). At the dose utilized, mice exhibit significant morbidity at days 3 to 6 and recovery occurs at days 7 to 11. Serum was isolated at specific intervals of the infection to query for evidence of complement activation. The central complement protein C3 is activated by proteolysis to produce the soluble anaphylatoxin C3a (18). The concentration of C3a in serum was measured by enzyme-linked immunosorbent assay (ELISA) to assess the level of *in vivo* complement activation. As a positive control, naive C57BL/6J mouse serum (B6MS) was incubated with the insoluble yeast polysaccharide and potent complement activator zymosan (zB6MS) (19). As shown in Fig. 1A, zB6MS activation resulted in a significant increase in the concentration of C3a compared to B6MS. Analysis of serum C3a concentration from R. australis-infected animals exhibited elevated C3a concentration at day 3 postinfection, revealing that complement is indeed significantly activated in experimentally infected animals.

To examine if C3a production is a direct consequence of Rickettsia interaction with the complement system, we incubated purified bacteria with B6MS. As a positive control, serum was again activated with zymosan (zB6MS). As shown in Fig. 1B, incubation of mouse serum with zymosan leads to production of C3a. Similarly, C3a production increased after incubation of R. australis with B6MS but not with phosphate-buffered saline (PBS). This result demonstrates that complement is directly activated by exposure to R. australis.

Since the complement system is directly activated by R. australis exposure, we examined if this phenotype had any effect on viability of the bacteria. To this end, R. australis and serum-sensitive Escherichia coli were incubated under the same conditions that resulted in complement activation (Fig. 1B). The quantity of live bacteria remaining after PBS incubation or serum challenge was determined by titration (R. australis) or limiting dilution plating (E. coli). Serum-borne complement is able to effectively kill E. coli but is not capable of eliminating R. australis from serum (Fig. 1C). This result demonstrates that, like other pathogenic rickettsial species, R. australis is resistant to bacteriolytic complement attack in naive serum (14–16, 20) and that these bacteria are capable of acquiring the serum regulator vitronectin (see Fig. S1 in the supplemental material). These results also suggest that *in vivo* complement activation may not have a directly detrimental effect on bacterial viability.

### Functional complement is essential for the immune response to R. australis infection.

**FIG 2:**
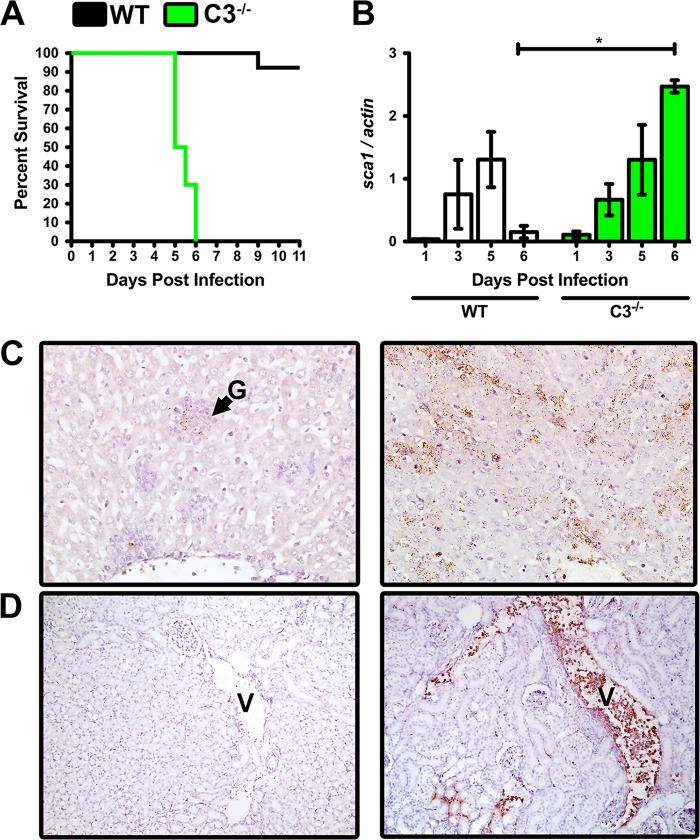
Intact complement is required for control of a normally sublethal R. australis infection. (A) Survival of WT mice (black) and mice containing a targeted mutation of the central complement protein C3 (C3^−/−^) (green) after challenge with a sublethal dose of intravenous R. australis. (B) Preidentified mice were removed from each experimental group at 1, 3, 5, and 6 days postinfection. The quantity of bacteria in the infected liver was determined by analysis of the ratio of *R. australis sca1* to *M. musculus actin* DNA. (C) Immunohistochemical (IHC) analysis of rickettsial antigen (brown) in the R. australis-infected liver at day 6 postinfection in WT (left panel) and C3^−/−^ (right panel) mice. Pyogranuloma is designated by “G” (magnification, ×40). (D) IHC analysis of day 6 kidney in R. australis-infected WT (left panel) and C3^−/−^ (right). Renal venules are annotated with a “V” (magnification, ×20). *, *P* < 0.05.

The findings shown in Fig. 1 describe a contradictory dichotomy whereby R. australis readily activates the complement system *in vivo* and *in vitro* but the complement system does not directly affect bacterial fitness. These findings suggest two potential outcomes when examining the *in vivo* relationship between Rickettsia and complement: R. australis may be resistant to complement *in vivo*, or complement may contribute to anti-Rickettsia immunity in a manner that cannot be readily reproduced *in vitro*. To resolve this dichotomy, we challenged wild-type (WT) C57BL/6J mice and mice containing a targeted mutation in the *c3* gene with a normally sublethal dose of R. australis. C3^−/−^ mice are able to initiate complement but lack amplification apparatuses and all major complement effector mechanisms (8). R. australis-infected mice were monitored for clinical signs of disease until recovery or succumbing to the infection. WT mice experienced significant morbidity, but 9 of the 10 WT mice were able to control the infection and recovered normally (Fig. 2A). In contrast, all 10 C3^−/−^ animals succumbed to the infection at days 5 to 6 postinfection. This stark contrast in survival demonstrates that the mammalian complement system is required for the successful control of Rickettsia infection.

To examine if the complement system has a direct effect on the infection load of R. australis, we examined the bacterial DNA content in liver throughout the time course of infection. Three preidentified animals from each group were removed from the study at days 1, 3, 5, and 6 postinfection. After genomic DNA extraction, a single chromosomal DNA target was amplified for R. australis (*sca1*) and M. musculus (actin). WT and C3^−/−^ animals experienced similar kinetics of bacterial proliferation in the infected liver from days 1 to 5. However, there is a steep decline in the bacterial load in the WT animals at day 6 that is not apparent in the C3^−/−^ animals (Fig. 2B).

To confirm the differences in bacterial burden between WT and C3^−/−^ mice and to define injuries associated with infection, we processed day 6 mouse organs for anti-R. australis immunohistochemical (IHC) stain (brown) and pathological analyses. IHC analysis of the WT liver demonstrated little rickettsial antigen and limited inflammation, but a few bacilli and a pyogranuloma (G) are apparent (Fig. 2C, left). In contrast, the C3^−/−^ liver shows a marked increase in R. australis quantity in the capillary endothelium, Kupffer cells, and hepatocytes, with necrosis (Fig. 2C, right). The WT day 6 kidney had very few apparent bacteria, but a C3^−/−^ renal venule (V) was extremely bacteremic, with numerous intramonocytic cytoplasmic bacteria and additional bacteria within the interstitium of glomeruli (Fig. 2D). Additional IHC and pathological analysis indicated that the endothelium of the spleen, heart, and testes of C3^−/−^ animals were heavily infected (see Fig. S2 in the supplemental material). Significant necrosis was noted in tissues of the lymphatic system, with the exception of largely uninfected splenic white pulp. The overall difference in bacterial burden between WT and C3^−/−^ animals correlates strongly with the increased morbidity and mortality observed in the C3^−/−^ animals at days 5 to 6 postinfection. We therefore conclude that the mammalian complement system plays an indispensable role in controlling infection with the obligate-intracellular bacterium R. australis.

### The bacteriolytic portion of the complement system does not contribute to immunity.

**FIG 3:**
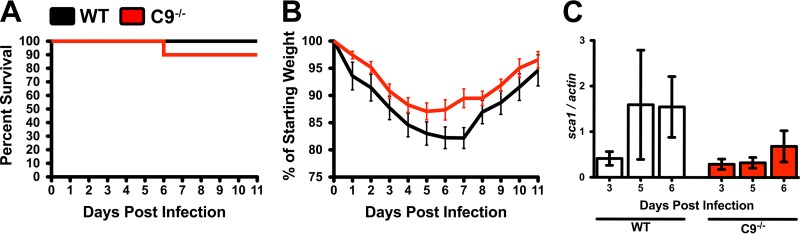
The complement-dependent membrane attack complex (MAC) does not contribute to control of R. australis infection. (A) Survival of WT mice (black) and mice containing a targeted mutation of the gene encoding complement protein C9 (red) upon challenge with a sublethal dose of intravenous R. australis. (B) Weight loss (morbidity) and recovery in WT (black) and C9^−/−^ (red) mice after infection. (C) PCR quantification of the ratio of *R. australis sca1* to *M. musculus actin* DNA in the liver of WT or C9^−/−^ animals.

We have previously established that Rickettsia species are resistant to the antibacterial effects of complement *in vitro* (15, 16). However, in this work, we establish that the complement system is both activated and essential for immunity to R. australis infection. We therefore queried whether the antibacterial membrane attack complex contributes to control of infection *in vivo*. To this end, we infected mice containing a targeted mutation in the *c9* gene. MAC pore is assembled by initial deposition of a lipophilic complex of C5b, C6, C7, and C8 followed by successive additions of multiple C9 peptides to form the lytic pore (21). As such, genetic ablation of the gene encoding C9 disrupts the formation of the MAC. However, because C9 is required only for the terminal complement activity, removal of the *c9* gene does not disrupt the remainder of the complement system. Observation of mice over the time course of infection demonstrated that both WT and C9^−/−^ mice were able to control the sublethal R. australis dose (Fig. 3A) with similar morbidity (weight loss) during the time course of infection (Fig. 3B).

To examine if C9 deficiency has any effect on R. australis pathogenesis, we examined the concentration of bacteria in the infected liver over the time course of the infection. Analysis of the ratio of Rickettsia (*sca1*) to murine (actin) DNA indicated no significant differences in the quantity of bacteria during infection (Fig. 3C). Neither mouse morbidity nor R. australis proliferation rates were significantly different during infection in WT and C9^−/−^ mice. These findings establish that the antibacterial MAC is dispensable for control of R. australis infection. Additionally, since the complement system is activated *in vivo* (Fig. 1A) but does not directly affect R. australis pathogenesis (Fig. 3), we conclude that R. australis is resistant to the lytic effects of complement *in vivo*, effectively confirming our previous findings (15, 16).

### Complement-dependent opsonophagocytosis is dispensable for the effective immune response.

R. australis is a potent complement activator (Fig. 1) but is unaffected by the antibacterial membrane attack complex *in vitro* and *in vivo* (Fig. 3). The other antibacterial activity associated with the complement system is opsonophagocytosis. The activated complement proteins C3b, C4b, and C5b (and their breakdown products) are the classical complement opsonins (22). Phagocytosis of opsonized particles is induced by interaction with cognate complement receptor (CR) proteins on the surface of various leukocytes.

**FIG 4:**
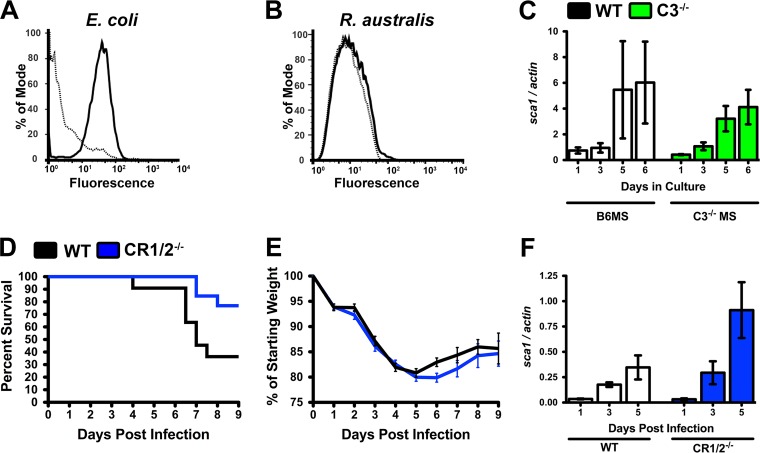
Complement-dependent opsonophagocytosis is dispensable for immunity to R. australis infection. (A, B) Flow-cytometric analysis of deposition of the C3b opsonin on E. coli (A) and R. australis (B) after incubation in PBS (dotted line) or normal mouse serum (NMS) (solid line). (C) R. australis growth in murine macrophage RAW264.7 cells after preincubation with B6MS (white) or C3^−/−^MS (green). (D) Survival of WT (black) and CR1/2^−/−^ (blue) mice after intravenous challenge with R. australis. (E) Weight loss (morbidity) after R. australis infection in WT (black) and CR1/2^−/−^ (blue) mice. (F) PCR analysis of the ratio of *R. australis sca1* to *M. musculus actin* DNA in the infected liver of WT and CR1/2^−/−^ mice at specific time points postinfection.

To determine if complement opsonization occurs on the R. australis surface, we incubated bacteria with murine serum under the same conditions that lead to complement activation (Fig. 1B) and employed flow cytometry to query for potential deposition of the central complement opsonin C3b. As shown in Fig. 4A, deposition of C3b is apparent on serum-sensitive E. coli, but the lack of fluorescent change for R. australis indicates that C3b is not deposited on the surface of R. australis (Fig. 4B). In a parallel examination of complement-mediated opsonization, R. australis was preincubated with C57BL/6J mouse serum (B6MS) or C3^−/−^MS to allow for potential complement-mediated opsonization. The potentially opsonized Rickettsia bacteria were applied to RAW264.7 murine macrophages, a cell line that has the ability to phagocytose opsonized particles (23). Because there is no difference in the growth kinetics of R. australis after incubation in the two serum types (Fig. 4C), we conclude that complement opsonization does not affect growth in macrophage cells.

To assess if complement-mediated opsonization contributes to the immune response against R. australis infection, we infected mice with a targeted mutation in the *cr2* gene (24). The single *cr2* gene encodes two mature surface receptors, complement receptor 1 (CR1, CD35) and complement receptor 2 (CR2, CD21). CR1 is found on the surface of neutrophils, macrophages, and B cells and functions as a phagocytosis-promoting receptor (25). CR2-mediated signaling promotes B-cell activation through recognition of the C3b breakdown peptides (26). WT C57BL/6J and CR1/2^−/−^ mice were infected with an intravenous dose of R. australis. As shown in Fig. 4D, 80% of the CR1/2^−/−^ mice survived the infection compared to 40% of WT animals, indicating that CR1/2^−/−^ mice do not have increased susceptibility to infection. Similarly, infection-associated morbidity is not different between WT and CR1/2^−/−^ mice (Fig. 4E). Analysis of the ratio of R. australis to Mus musculus DNA within the infected liver also did not demonstrate any significant differences in bacterial burden (Fig. 4F). Since infection in CR1/2^−/−^ mice did not demonstrate increased morbidity or rickettsial load, we conclude that CR1/2^−/−^ mice do not have a deficient immune response to Rickettsia infection. Thus, there is no apparent contribution of complement-mediated opsonophagocytosis to the effective immune response to R. australis infection.

### Antibody-mediated immunity to R. australis is complement dependent.

**FIG 5:**
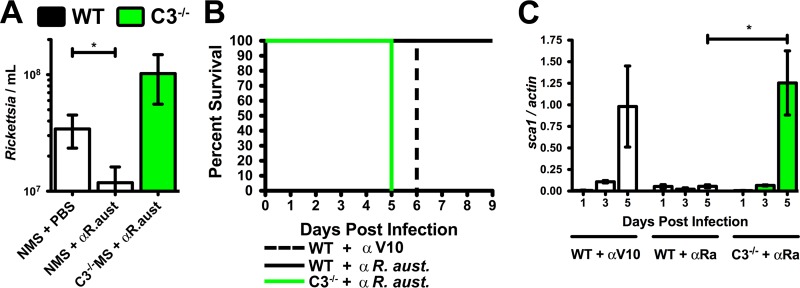
Complement is required for antibody-mediated immunity to R. australis infection. (A) *In vitro* complement-mediated killing of complement-resistant R. australis (normal mouse serum [NMS] + PBS) after addition of anti-R. australis polyclonal antibodies (NMS + αR. aust.). Antibody-enhanced killing is complement dependent as demonstrated by the loss of serum lethality in C3^−/−^MS. (B) Survival of animals after prophylactic treatment with anti-R. australis antibody (solid line) or irrelevant antibody (dashed line) and challenge with 4 minimum lethal doses of R. australis. Anti-R. australis-mediated protection fails in C3^−/−^ mice lacking a functional complement system (green line). (C) PCR analysis of the *Rickettsia sca1* to murine actin DNA ratio in the liver over the time course of infection in mock-treated (WT + αV10), antibody-protected (WT + αRa), and failed antibody protection (C3^−/−^ + αRa) mice. *, *P* < 0.05.

Previous studies have demonstrated that specific anti-Rickettsia antibodies can decrease infectious burden and temper disease (27–30). To assess the relationship between antibody-mediated immunity and complement functionality, we isolated serum immunoglobulin from C57BL/6J mice that had been infected with a sublethal dose of R. australis and subsequently recovered from infection. Addition of purified anti-R. australis antibodies to naive mouse serum can overcome the inherent *in vitro* complement resistance of R. australis (Fig. 5A). However, *in vitro* antibody-mediated killing of R. australis is complement dependent, as antibody-enhanced killing of R. australis is lost in serum lacking a functional complement system (C3^−/−^MS).

To determine if this *in vitro* phenotype occurs within the infected animal, we injected serum-free anti-R. australis antibodies into naive WT and C3^−/−^ mice and subsequently challenged the mice with 4 minimum lethal doses of R. australis (Fig. 5B). WT mice that were prophylactically treated with the anti-R. australis antibodies were protected from 4 minimum lethal doses of R. australis (Fig. 5B, solid line), whereas mice that were treated with an irrelevant antibody (anti-Yersinia pestis V10) succumbed to the infection (dashed line). However, antibody-mediated protection is dependent on a functional complement system because anti-R. australis-treated C3^−/−^ mice were unable to successfully combat the infection (Fig. 5B, green line). Analysis of the rickettsial load in the liver demonstrates a dramatic decrease in the quantity of bacteria in the anti-R. australis-protected mice at day 5 postinfection (Fig. 5C), which is not apparent in the other treatment groups. We therefore conclude that anti-Rickettsia antibodies can function by activating the complement system and that antibody-mediated immunity to R. australis infection requires a functional complement system.

### Phenotypes associated with loss of complement function implicate anaphylatoxin signaling in immunity.

We have established that a functional complement system is essential for the successful immune response to R. australis infection (Fig. 2), but the two direct killing mechanisms of the complement system are dispensable for immunity in naive animals (Fig. 3 and 4). The remaining portions of the complement system are thoroughly integrated into initiation and control of secondary immune mechanisms through anaphylatoxin signaling (8). The major pathological differences between WT and C3^−/−^ mice are observed during induction of the early adaptive immune response (days 5 to 6) (31). The temporal correlation between the observable immune defects and initiation of the adaptive response led to the hypothesis that the complement system contributes to R. australis immunity through anaphylatoxin signaling to amplify the effects of other immune mechanisms.

**FIG 6:**
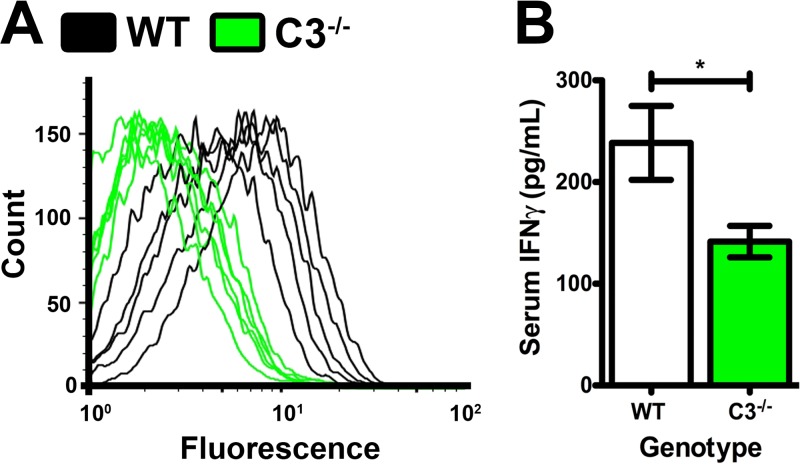
Identification of aberrant immune phenotypes in susceptible C3^−/−^ mice. (A) Flow-cytometric analysis of anti-R. australis IgG in WT (black) or C3^−/−^ (green) serum at day 6 postinfection. (B) Concentration of IFN-γ in serum at day 3 following R. australis infection in WT (white) and C3^−/−^ (green) animals. *, *P* < 0.05.

To explore this hypothesis, we examined the quantity of anti-Rickettsia antibodies present in WT and C3^−/−^ animals at day 6 postinfection. Flow-cytometric analysis of IgG recognition of the bacterial surface demonstrated that WT mouse serum contained anti-Rickettsia IgG (Fig. 6A, black traces). Conversely, day 6 C3^−/−^ serum contained less IgG that recognized the bacterial surface. These data intimate that Rickettsia-induced complement activation increases the production of IgG antibodies in infected mice. In addition, analysis of serum levels of the cytokine gamma interferon (IFN-γ) in WT and C3^−/−^ mice at day 3 postinfection demonstrates a significant decrease in IFN-γ in mice lacking a functional complement system (Fig. 6B). Since IFN-γ production correlates strongly with the effective immune response to rickettsial infections (32), we posit that the complement system contributes to the innate immune response to R. australis infection through induction of IFN-γ production. Finally, pathological analysis of WT and C3^−/−^ mice at day 6 postinfection demonstrated a marked increase in spleen hyperplasia in the WT mice, demonstrating that complement functionality correlated with induction of the adaptive immune response (see Fig. S3 in the supplemental material). Loss of complement functionality in C3^−/−^ mice results in reduced IFN-γ production, IgG secretion, and induction of the adaptive response. Together, these phenotypes implicate anaphylatoxin signaling as the effective portion of the complement system during R. australis infection.

## DISCUSSION

Here, we have analyzed the relationship between the host complement system and an obligate intracellular pathogen. A recent report demonstrated that patients undergoing infection with the obligate intracellular bacterium R. conorii present with elevated levels of activated complement proteins (13). Additionally, rickettsial species express surface proteins that are sufficient to mediate resistance to the antibacterial effects of serum complement (14–16, 20). We reasoned that while pathogenic Rickettsia species can activate complement, this class of pathogens have evolved mechanisms to resist complement-mediated killing during infection. Indeed, we determined that complement was activated *in vivo* following intravenous R. australis infection of B57BL/6J mice. Additionally, complement activation is essential for the immune response to Rickettsia infection, as mice lacking the central complement protein C3 were more susceptible to infection than their WT counterparts. The impact of complement also extends to antibody-mediated immunity to Rickettsia infection, because prophylactic protection with anti-R. australis antibodies fails in the absence of functioning complement.

To identify the mechanism of complement effectiveness, we utilized a series of mice lacking individual complement-dependent effector apparatuses. C9^−/−^ mice lack the ability to produce the antibacterial porelike membrane attack complex (MAC). Analysis of infection in these mice indicated that C9 is completely dispensable for immunity and that the MAC plays no role in controlling Rickettsia infection. Complement activation can also result in opsonization and phagocytic removal of a target particle. Flow-cytometric analysis of deposition of the opsonin C3b indicated that R. australis is not readily opsonized *in vitro*, and infection of mice lacking complement receptors indicated that opsonophagocytosis does not contribute to immunity. We did, however, observe that IFN-γ production, IgG secretion, and spleen hyperplasia were decreased in susceptible animals lacking the entire complement system. These data indicate a correlation between complement activation and induction of secondary immune effectors.

The results described above were obtained from manipulation of the host immune system. The experiments were designed to directly assess the genetic requirements for the immune response to this pathogen. However, the results can also be interpreted from the Rickettsia pathogenesis point of view. The complement system is activated during an ongoing Rickettsia infection in humans and mice (13). Complement activation implies that the complement system is aggressively attempting to clear the bacteria through the innate antibacterial complement mechanisms. And yet, R. australis is resistant to the effects of the MAC and complement-mediated opsonophagocytosis. R. australis-encoded MAC and opsonization evasion mechanisms are indeed contributing to pathogenesis. The results have identified a fundamental aspect of rickettsial pathogenesis and also suggest that these virulence processes are a target for future therapeutic intervention. The lack of MAC and opsonophagocytosis efficacy demonstrates that Rickettsia actively evades the two antibacterial components of the complement system. However, these complement resistance phenotypes are not without cost to the bacterium. Rickettsia complement resistance is associated with activation of the signaling components of the complement system, and complement-mediated enhancement of secondary immune mechanisms appears to contribute to more-effective clearance of the pathogen.

The mechanism(s) by which rickettsial species and potentially other obligate intracellular pathogens evade complement-mediated opsonophagocytosis is not yet elucidated. Because the complement system exists almost exclusively outside the host cell, few studies have been conducted to determine the connection of this arm of the immune system to intracellular bacteria. However, clinical data demonstrated that the complement system is activated during R. conorii infection (13). A few experimental analyses have established complement efficacy against facultative or obligate intracellular bacteria; removal of complement increases lung infectivity of Chlamydia psittaci, and complement receptor-dependent entry of Mycobacterium leprae leads to intracellular killing (33, 34). Together, these studies suggest that the complement system may have a more universal effect on obligate intracellular bacteria.

Our results clearly demonstrate that R. australis is not readily opsonized by professional phagocytes, suggesting that R. australis possesses the ability to inhibit C3b deposition or to increase the rate of C3 turnover. Rickettsia acquisition of factor H might conceivably play a role in this phenotype (15), as factor H serves as a competitive inhibitor of the C3 convertase and as a cofactor for degradation of C3b. However, rickettsial acquisition of factor H would have little influence on classical or lectin pathways of complement activation, as factor H has a limited role in regulating these complement activation pathways. The current data do not rule out other rickettsial mechanisms of decreasing opsonization, including complement-targeted protease production, complement regulator mimicry, or acquisition of other complement regulator proteins (35). A recent publication described the identification and characterization of a rickettsial HIV-1 like protease (RC1339/APRc) that is expressed at the outer membrane of two related species, R. conorii and R. rickettsii (36). While this protein is sufficient to proteolytically process two conserved rickettsial autotransporter proteins, Sca0/OmpA and Sca5/OmpB, very little else is known about putative additional functions for APRc and APRc homologues in other rickettsial species, including R. australis (WP_014412171). Whether APRc or other putative secreted proteases are involved in the resistance to complement-mediated killing warrants further investigation.

Analysis of the contribution of the antibacterial complement effectors has also increased understanding of the molecular mechanism of rickettsial resistance to the antibacterial effects of the C9-dependent membrane attack complex (MAC). E. coli is susceptible to complement-mediated killing in naive serum, but Rickettsia is resistant to this immune clearance mechanism. The Rickettsia integral outer membrane proteins Adr1 and Adr2 have been demonstrated to mediate acquisition of the soluble complement-regulatory protein vitronectin (14, 16). R. australis encodes Adr1 and Adr2, with 71% and 95% similarity between R. conorii and R. australis amino acid sequences, respectively. R. australis Adr1 and Adr2 proteins are annotated as WP_014412225.1 and WP_014412224.1. Importantly, these genes maintain genomic linkage with the two open reading frames next to each other, suggesting that R. australis encodes proteins capable of acquiring vitronectin from serum, thus inhibiting MAC-mediated bacterial lysis (Fig. S1) (37).

Loss of complement functionality in C3^−/−^ mice results in decreased *in vivo* IFN-γ production, reduced IgG secretion, and lack of induction of the adaptive response, suggesting that complement-mediated signaling activities are the portion of the complement system that contributes to an effective immune response to this pathogen. The complement anaphylatoxins, C3a and C5a, and to a lesser extent C4a, are important modulators of the immune response to infection. These small proteins are produced during complement activation and are detected by the G-protein-coupled receptors C3aR and C5aR (38). C3aR- and C5aR-dependent signaling induces a variety of physiological responses, including respiratory burst in phagocytes, endothelial cell activation, histamine production, cytokine production, Th1 skew, T-cell survival, and B-cell activation (reviewed in reference 8). Infection of mice lacking C3 generates phenotypes associated with loss of anaphylatoxin signaling, including decreased cytokine production, decreased IgG secretion, and a reduced proliferation of lymphocytes in the spleen. We therefore hypothesize that the complement system contributes to the effective immune response to Rickettsia infection through anaphylatoxin signaling mechanisms. Genetic ablation of the anaphylatoxin receptors in the mouse is possible, but the genetically modified mice are not readily available (39). As such, analysis of Rickettsia infection and the subsequent immune response in mice lacking anaphylatoxin receptors is currently an active area of investigation.

Recent scientific and regulatory developments have led to FDA approval of the first set of complement inhibitors for use in treating genetic disorders. Additional complement-inhibiting drugs are proceeding through the FDA approval process, and existing drugs are being repurposed for use in more-common diseases, including arthritis, and in transplantation and hemodialysis patients (40). The use of complement-inhibiting medicine conflicts with our finding that mice lacking complement effectors are acutely susceptible to Rickettsia infection. Application of our data to human infections suggests that patients treated with complement-inhibiting drugs may have increased susceptibility to Rickettsia infection. A similar phenotype has been noted for patients receiving the drug eculizumab (Soliris) and having increased susceptibility to meningococcal infection (41). We fear that a similar risk exists with Rickettsia infections, but a correlation would be identified only after patients have experienced negative effects.

We have shown that R. australis is capable of inducing the activation of complement in serum; however, there is very little known regarding the putative mechanism(s) by which this class of obligate intracellular pathogens directly or indirectly stimulates complement activity. There are three known mechanisms of complement activation: (i) the classical pathway (CP), (ii) the lectin pathway (LP), and (iii) the alternative pathway (AP). The CP is activated by deposition of a single IgM or multiple IgG molecules on a target surface (42). Other studies also indicate that the CP protein C1q can interact directly with C-reactive protein, lipopolysaccharide (LPS), and bacterial porins (43, 44). Rickettsia does produce LPS and encode porins (45, 46), but whether these are involved in the complement activation pathway has yet to be explored. Interestingly, convalescent-phase serum, but not naive serum, is able to directly lyse and opsonize Rickettsia, presumably through CP activation (27, 47). The LP is activated by deposition of the soluble pattern recognition receptors mannose binding lectin, collectins, and ficolins (2, 48). A recent genetic analysis indicated that Rickettsia spp. likely synthesize and integrate UDP-*N*-acetyl-d-mannoseamine into LPS (49), thus providing a potential substrate for mannose binding lectin. However, this potential interaction necessitates further experimental investigation. Finally, complement can be activated through the alternative pathway (AP). The AP monitors for pathogens by maintaining low-level constitutive activation and covalent linkage of C3b-H_2_O to a free hydroxyl group on the target surface (50). In the absence of specific regulators commonly found on healthy mammalian cells, complement will be exponentially activated, leading to an inflammatory response and attempted pathogen clearance. Molecular evidence suggests that Rickettsia may be at least partially resistant to AP activation by acquisition of the serum complement-regulatory protein factor H by the conserved porin-like C-terminal OmpB translocon domain (OmpB βp) (15). Future assessment of the utility of exogenous complement induction in combating Rickettsia infection will potentially identify a novel strategy for treatment of these significant infections.

In summary, we have utilized *in vitro* and *in vivo* models of infection to define the interplay between pathogenic Rickettsia australis and the murine complement system. Genetic elimination of the complement system increases susceptibility of mice to R. australis infection. Antibody-mediated immunity to this pathogen also requires an intact complement system. However, increased susceptibility to Rickettsia infection is not observed in mice lacking the two main complement antibacterial mechanisms, opsonization and phagocytosis. Instead, phenotypes associated with complement-mediated immune signaling are observed in susceptible animals, suggesting that anaphylatoxin signaling is a mediator of complement-dependent immunity to Rickettsia infection. These findings have established an experimental system for analyzing the influence of anaphylatoxin signaling during an infection lacking other functioning complement mechanisms. Importantly, these data demonstrate that the complement system can have a powerful and essential effect on obligate intracellular infections. A more complete understanding of this interaction may identify new roles for complement and for potential complement-targeted therapeutic intervention against rickettsial species and other obligate intracellular pathogens.

## MATERIALS AND METHODS

### Cell lines.

Rickettsia australis was routinely cultured in Vero cells (ATCC CCL-81) in Dulbecco's modified Eagle medium (DMEM) with 10% fetal bovine serum, nonessential amino acids, and sodium pyruvate. R. australis was additionally cultured in RAW 264.7 cells (ATCC TIB-71) in RPMI with 10% fetal bovine serum.

### Animal husbandry.

The following murine models were employed in this study: (i) C57BL/6J mice; (ii) for complement component C3-targeted mutations, B6;129S4-C3^tm1Crr^/J mice (C3^−/−^) (4), (iii) for complement component C9-targeted mutation, B6N(Cg)-C9^tm1.1(KOMP)Vlcg^/J (C9^−/−^) mice (51); and (iv) for complement receptor 1/2 (*cr2^−/−^*)-targeted mutation, B6.129S7(NOD)-CR2^tm1Hmo^/J (CR1/2^−/−^) mice (24). Mice were used as homozygotes, and genotypes were confirmed according to the appropriate genotyping PCR protocol (Jackson Laboratories). All infected mice were utilized at 5 to 7 weeks of age and were euthanized by 11 weeks old. Wild-type (WT) C57BL/6J animals were acquired from Jackson Laboratory and were age and sex matched to the genetically modified groups.

### Animal models.

The R. australis quantity was determined by titration as has been previously described (52). We previously determined that the minimal lethal dose of R. australis by intravenous retro-orbital inoculation was 1 × 10^6^ bacteria per 5- to 7-week-old C57BL/6J (wild-type) mouse. Accordingly, the sublethal dose utilized here was 5 × 10^5^ bacteria/mouse. At this dose, wild-type mice demonstrate significant morbidity but generally do not succumb to infection. The sublethal dose was utilized for examining potential defects in the ability to control R. australis infection in C3^−/−^, C9^−/−^, and CR1/2^−/−^ mice. For comparison of infections in different mouse genotypes, all groups were age and sex matched.

To isolate anti-R. australis immune serum, 13 5- to 7-week-old WT mice were infected with a sublethal dose of R. australis, and serum was recovered from euthanized mice after full recovery at day 14 postinfection. The sera were pooled, diluted in 0.02 M sodium bicarbonate (pH 8) buffer, and filtered to remove any infectious agents. Total antibodies were isolated using a 5-ml HiTrap protein G column (GE Health Care Biosciences) and dialyzed into a phosphate-buffered saline (PBS) solution. Fifty micrograms of anti-R. australis or irrelevant antibody (anti-Y. pestis V10) (53) was injected into the mouse peritoneum 30 min before retro-orbital inoculation with 4 minimum lethal doses (4 × 10^6^ cells) of R. australis.

For all infections, 22 5- to 7-week-old WT and targeted mutant mice were inoculated by retro-orbital injection with R. australis. Infected mice were monitored twice daily for signs of disease and daily for weight change. Ten animals were monitored until they recovered or exhibited signs consistent with succumbing to infection. These mice were removed from the study and scored as succumbing to the infection as has been previously described (27). Overt clinical signs of R. australis infection included ruffled fur, hunched posture, shallow respiration, immobility when touched, and weight loss of at least 15% of initial body weight. The remaining 12 mice were utilized to examine the time course of infection, whereby 3 predesignated mice were euthanized at 1, 3, 5, and 6 days postinfection (when surviving animals remained). For each animal, the spleen, kidney, liver, heart, lung, and blood were aseptically extracted and split into sections for use in PCR analyses, serum analysis, and pathological examination.

### Serum samples.

Serum samples described as normal mouse serum (NMS) and activated zymosan were purchased from Complement Technologies. C57BL/6J blood was isolated by cardiac puncture, and the samples were processed with Z-gel (Sarstedt) to recover serum. All serum was snap-frozen and stored at −80°C when appropriate. Zymosan-activated C57BL/6J mouse serum (zB6MS) was generated by addition of 1:20 activated zymosan (Comptech), followed by incubation at 37°C for 1 h.

### Serum resistance.

R. australis or E. coli DH5α cells (1 × 10^6^) were incubated with 50% NMS for 1 h at 34°C. The quantity of input and surviving bacteria was determined by titration (R. australis) or CFU on LB agar (E. coli). To assess antibody-mediated immunity, a mixture of 1.2 × 10^4^ cells of R. australis, 50% C57BL/6J or C3^−/−^ serum, and 12.5 μg of mouse anti-R. australis or anti-Y. pestis V10 polyclonal antibody was incubated at 34°C for 1 h with agitation. After incubation, all samples were immediately placed on ice to inhibit complement activity, and remaining bacteria were quantified by titration.

### Indirect immunohistochemistry.

Murine tissues were collected immediately after euthanasia and fixed in 10% buffered formalin (1:10 tissue-formalin ratio). Samples were routinely processed and embedded in paraffin, and 5-μm sections were cut for hematoxylin and eosin (H&E) staining. Isolated tissues were additionally examined by immunohistochemistry to localize R. australis within the infected animals using anti-RcPFA, which recognizes several SFG rickettsial species (27). Primary antibody staining was followed by biotinylated anti-rabbit IgG secondary antibody (1:1,000, vector BA 1000; Vector Laboratories) and exposure to the detection reagent (Vectastain ABC rabbit IgG kit, vector PK 6102; Vector Laboratories). Slides were analyzed, micrographs captured, and pathological changes recorded by a Diplomate of the American College of Veterinary Pathologists (DACVP).

### Flow cytometry.

For analysis of *in vivo* IgG production in response to R. australis infection, paraformaldehyde (PFA)-fixed R. australis was incubated with 1:25 serum from WT or C3^−/−^ mice at 6 days postinfection. The solution was incubated at 34°C for 1 h with agitation, washed with PBS, and fixed with 4% PFA. Mouse IgG deposition on the R. australis surface was determined by incubation with 1:500 goat anti-mouse IgG-Alexa Fluor 488 and flow cytometry on a FACSCalibur instrument. For analysis of C3 deposition on the bacterial surface, E. coli DH5α was grown overnight in LB broth at 37°C with agitation. Bacteria were sedimented by centrifugation and resuspended in 4% PFA. E. coli or unfixed R. australis bacteria were incubated with PBS or 50% naive C57BL/6J serum at 34°C for 1 h. After washing, all bacteria were fixed with 4% PFA and blocked with 2% bovine serum albumin in PBS. C3 deposition after naive serum incubation was determined with 1:100 rabbit anti-mouse C3 (PA5-21349; Thermo Fisher Scientific) and 1:500 goat anti-rabbit IgG-Alexa Fluor 488 followed by flow cytometry on a FACSCalibur instrument. Vitronectin deposition on R. australis was performed as previously described (16).

### ELISA.

For detection of murine C3a, MaxiSorp 96-well plates (Nunc) were incubated with 5 μg/ml of the capture antibody rat anti-mouse C3a (clone I87-1162; BD Pharmagen) overnight at 23°C. After blocking, 1:200 serum samples or mouse C3a protein (BD Pharmagen) was incubated for 1 h. C3a content was determined by sequential incubation with biotin rat anti-mouse C3a (clone I87-419; BD Pharmagen), 1 μg/ml streptavidin-horseradish peroxidase (Pierce), OptiEIA 3,3′,5,5′ tetramethylbenzidine (TMB) substrate (BD Pharmagen), and 2 M H_2_SO_4_. Murine IFN-γ concentration was determined by enzyme-linked immunosorbent assay (ELISA) according to the manufacturer's instructions (Neo Scientific). Absorbance was measured at 450 nm.

### Quantitative PCR.

Genomic DNA was isolated from murine liver or RAW 264.7 cells utilizing the PureLink genomic DNA 96-well kit (Invitrogen). The ratio of R. australis to M. musculus DNA was determined by quantitative PCR utilizing the TaqMan gene amplification master mix (Applied Biosystems) and a LightCycler 480 instrument (Roche). R. australis was detected using *sca1*_RA_5220F (5′-TGCAGAACAAGTTTGTTATTACCC-3′), *sca1*_RA_5465R (5′-CTACCGCTCCTTGGAACGTTAGACC-3′), and *sca1*_RA_probe (5′-56-FAM/TCGGCTTAA/Zen/GATATGGGAAGT/3IABlFQ/-3′). Murine actin primers and probes were previously described (54). All unknown samples were graphed against a standard curve of the specific amplicon cloned into pCR2.1. Data are expressed as the ratio of *R. australis sca1* to *M. musculus actin* DNA.

### Statistical analyses.

Comparison of serum C3a concentration in serum was performed by 1-way analysis of variance (ANOVA) with Dunnett's multiple comparison to control (B6MS). Survival was analyzed using comparison of survival curves with the log rank (Mantel-Cox) test. Analysis of DNA content in infected organs was analyzed by 1-way ANOVA with Bonferroni's multiple comparison of paired days. Serum survival was analyzed by 1-way ANOVA with Tukey's multiple comparison. Serum IFN-γ was analyzed by Student's *t* test.

### Ethics statement.

Animal experiments were conducted in accordance with protocols approved by the Institutional Biological and Recombinant DNA Safety Committee (IBRDSC) and Institutional Animal Care and Use Committee (IACUC) at the Louisiana State University School of Veterinary Medicine, protocol number 16-004. Standards of care and use for all animals conform to all applicable standards and regulations as established by the current version of the Animal Welfare Act and the *Guide to the Care and Use of Laboratory Animals*. This institution is fully accredited by the Association for the Assessment and Accreditation of Laboratory Animal Care, International (AAALAC), indicating verified compliance with the requirements for the proper care and treatment of all vertebrate laboratory animals, irrespective of species, location, investigator, use, or funding source. The University has on file with the Office of Laboratory Animal Welfare (OLAW) an approved Assurance Statement (number A3612-01).

## Supplementary Material

Supplemental material
