# Supplementary material for: Immunity against the Obligate Intracellular Bacterial Pathogen Rickettsia australis Requires a Functional Complement System
Source: Infect Immun. 2018 May 22;86(6):e00139-18. doi: 10.1128/IAI.00139-18 (PMC5964522; doi:10.1128/IAI.00139-18)
Supplement: Supplemental material [file supp_86_6_e00139-18__index.html]

Supplemental material 

# Immunity against the Obligate Intracellular Bacterial Pathogen Rickettsia australis Requires a Functional Complement System

## Supplemental material

- Supplemental file 1 -

  Fig. S1. Flow cytometric analysis of vitronectin acquisition by *R. australis*. Fig. S2. Immunohistochemical analysis of *R. australis* antigen in organs. Fig. S3. Pathological analysis of spleen.

  PDF, 4.5M
