# Supplementary material for: Immunity against the Obligate Intracellular Bacterial Pathogen Rickettsia australis Requires a Functional Complement System
Source: Infect Immun. 2018 May 22;86(6):e00139-18. doi: 10.1128/IAI.00139-18 (PMC5964522; doi:10.1128/IAI.00139-18)
Supplement: Supplemental material [file IAI.00139-18_zii999092417s1.pdf]

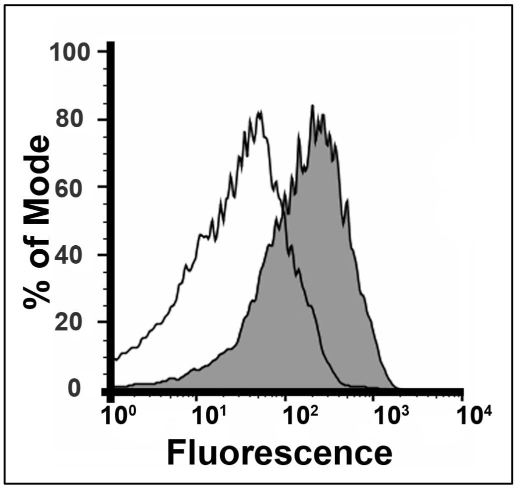

Figure S1. Flow cytometric analysis of vitronectin acquisition by *R. australis.* *R. australis* was incubated with PBS (white) or Normal Mouse Serum (gray). Vitronectin (Vn) deposition was assessed by anti-Vn antibody, fluorescent secondary antibody, and analysis by flow cytometry. The increase in fluorescence after incubation with serum indicates that *R. australis* is capable of binding mouse Vn.

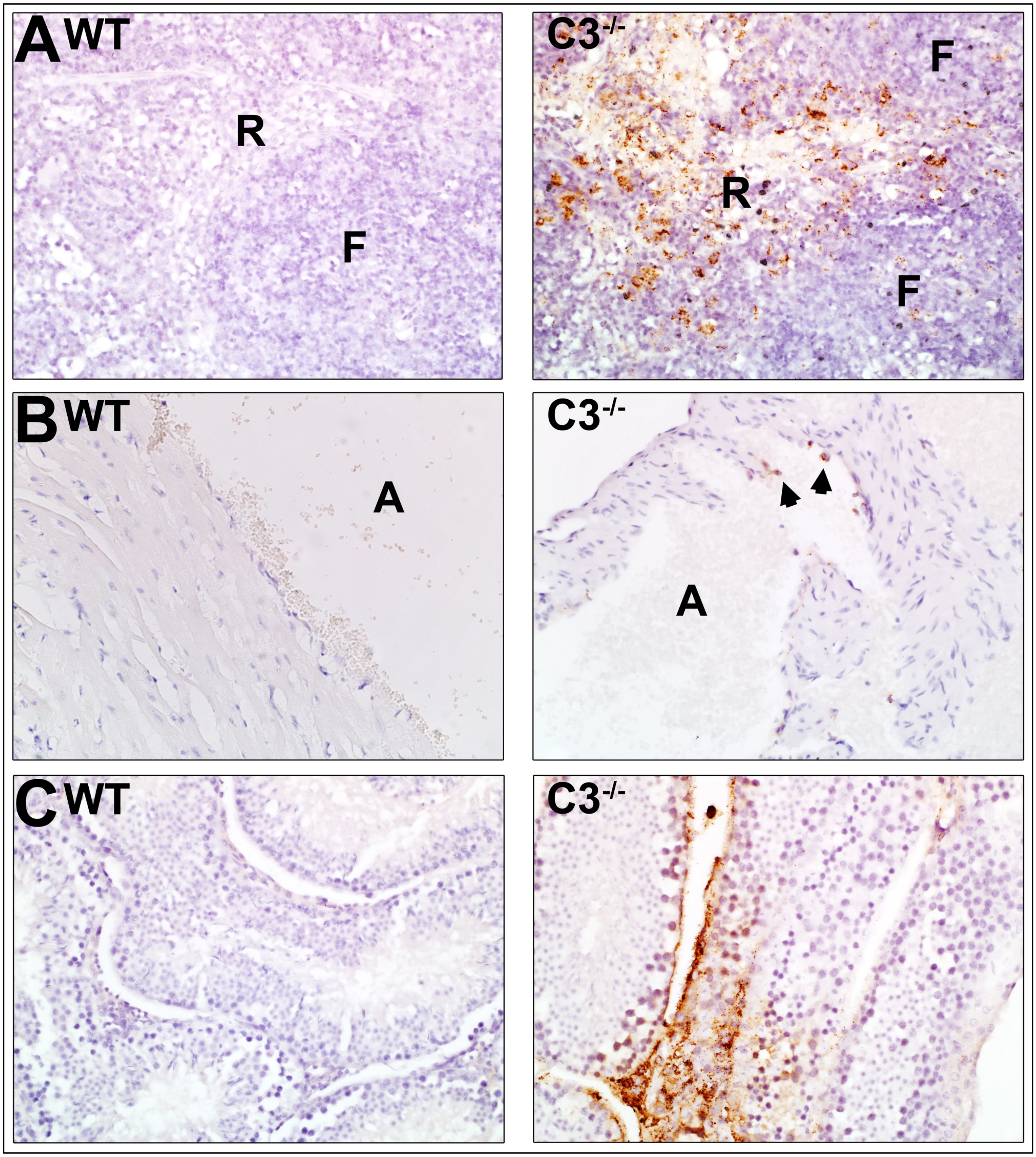

Figure S2. Immunohistochemical analysis of *R. australis* antigen in organs. Anti-*R. australis* immunohistochemical analysis of WT (left) and C3$^{-/-}$ (right) animals at D=6. **(A)** Spleen. WT= No antigen noted. C3$^{-/-}$= Numerous bacteria are gathering within the cytoplasm of histiocytes of the splenic red pulp and a few follicular tingible bodies macrophages contain intracytoplasmic bacteria. Follicles are designated by “F” and red pulp is designated by “R”.

40X. **(B)** Heart Atrium. WT= No bacteria and no lesions are present. $C3^{-/-}$= Bacteria within the endothelium cytoplasm and within the cytoplasm of monocytes marginating on the endothelial surface. Infected marginating lymphocytes are designated with arrows, and the atrium is designated with “A” No lesions were observed within the endothelium and cardiocytes. 40X. **(C)** Testes. WT= No bacteria and no lesions are present. $C3^{-/-}$= Numerous bacteria are within the cytoplasm of endothelial cells lining the seminiferous tubules. There are no histologic lesions. 40x.

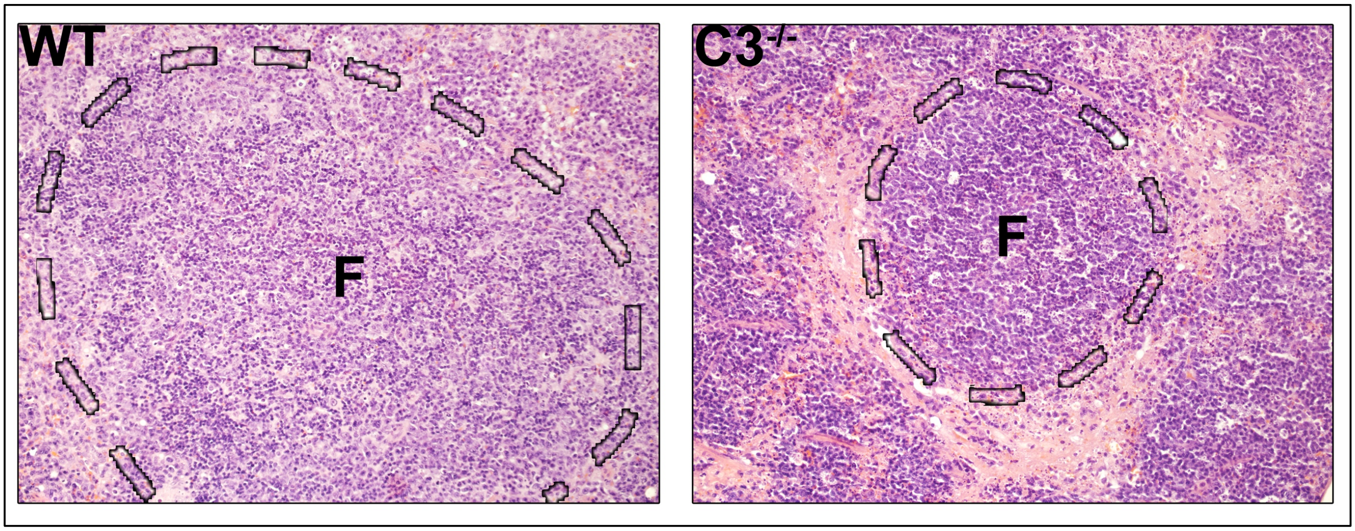

Figure S3. Pathological analysis of spleen. Hematoxylin and Eosin stain of WT (left) and $C3^{-/-}$ (right) spleen at D=6. WT= Reactive spleen with follicular (F) hyperplasia, lymphoplasmacytic and histiocytic infiltration of the red pulp. There is no evidence of necrosis. $C3^{-/-}$= Red pulp necrosis with fibrin and karyolysis, and intrafollicular tingible body macrophages containing intracytoplasmic leukocyte debris. Follicles are delineated with a “F” and outlined. 20X.
